# miR-25-3p promotes endothelial cell angiogenesis in aging mice via TULA-2/SYK/VEGFR-2 downregulation

**DOI:** 10.18632/aging.103834

**Published:** 2020-11-17

**Authors:** Chong Lian, Lei Zhao, Jiacong Qiu, Yang Wang, Rencong Chen, Zhen Liu, Jin Cui, Xiaonan Zhu, Xuejun Wen, Shenming Wang, Jinsong Wang

**Affiliations:** 1 Division of Vascular Surgery, The First Affiliated Hospital, Sun Yat-Sen University, Guangzhou 510080, China; 2 National-Local Joint Engineering Laboratory of Vascular Disease Treatment, Guangzhou 510080, China; 3 Guangdong Engineering and Technology Center for Diagnosis and Treatment of Vascular Diseases, Guangzhou 510080, China; 4 Department of Pharmacology Laboratory, Zhongshan School of Medicine, Sun Yat-Sen University, Guangzhou 510080, China; 5 Institute for Engineering and Medicine, Department of Biomedical Engineering, Chemical and Life Science Engineering, Virginia Commonwealth University, Richmond, VA 23284, USA

**Keywords:** aging, angiogenesis, endothelial cell, miR-25-3p, TULA-2

## Abstract

In aging, the regulation of angiogenesis is a dynamic and complex process. We aimed to identify and characterize microRNAs that regulate angiogenesis during aging. We showed that, in response to vascular endothelial senescence, microRNA-25-3p (miR-25-3p) plays the role of an angiogenic microRNA by targeting TULA-2 (T-cell ubiquitin ligand-2)/SYK (spleen tyrosine kinase)/VEGFR-2 (vascular endothelial growth factor receptor 2) signaling *in vitro* and *in vivo*. Mechanistic studies demonstrated that miR-25-3p inhibits a TULA-2/SYK/VEGFR-2 signaling pathway in endothelial cells. In old endothelial cells (OECs), upregulation of miR-25-3p inhibited the expression of TULA-2, which caused downregulation of the interaction between TULA-2 and SYK and increased phosphorylation of SYK Y323. The increased SYK Y323 phosphorylation level upregulated the phosphorylation of VEGFR-2 Y1175, which plays a vital role in angiogenesis, while miR-25-3p downregulation in YECs showed opposite effects. Finally, a salvage study showed that miR-25-3p upregulation promoted capillary regeneration and hindlimb blood flow recovery in aging mice with hindlimb ischemia. These findings suggest that miR-25-3p acts as an agonist of TULA-2/SYK/VEGFR-2 and mediates the endothelial cell angiogenesis response, which shows that the miR-25-3p/TULA-2 pathway may be potential therapeutic targets for angiogenesis during aging.

## INTRODUCTION

Aging is a pivotal risk factor for coronary artery disease, peripheral arterial occlusion and ischemic disease by affecting the physiological characteristics of arteries [1–3]. Angiogenesis is the formation of new blood vessels from the original vascular system, a multiplex process involving endothelial cell (EC) migration, activation, and proliferation [4]. Vascular regeneration can improve the prognosis of diseases in which vascular ECs play a key role in angiogenesis [5]. Previous studies have proved that the germination capability of collateral arterioles and capillaries declines in the lower extremities of aging animals [1, 2, 5].

MicroRNA (miRNA) plays a role in post-transcriptional regulation in gene expression. By binding to the bases of the genes, miRNAs can inhibit gene-to-protein translation, accelerate gene degradation, regulate gene expression and intracellular signaling pathways including the differential expression of angiogenesis-related proteins during aging [6].

The role of miRNAs in regulating EC protein expression and inducing changes in vascular endothelial function has attracted widespread attention from the cardiovascular community. Some changes in miRNA expression profiles are associated with decreased EC function [7], but details of the mechanisms remain unclear.

T-cell ubiquitin ligand-2 (TULA-2) belongs to the T-cell signaling protein (STS) /TULA family and is a protein tyrosine phosphatase (PTP) encoded by the UBASH3B gene that can be detected in the spleen, lung, liver, kidney and other tissues and cells, especially platelets [8, 9]. TULA-2 can negatively regulate the FcγRIIA-mediated platelet activation pathway receptor by dephosphorylating the Y323 site of spleen tyrosine kinase (SYK Y323) [10, 11]. SYK, as a nonreceptor tyrosine kinase, plays roles in physiological and pathological processes including angiogenesis, and has multiple phosphorylation sites [12]. The phosphorylation of SYK Y323 can activate protein phosphorylation of vascular endothelial growth factor receptor 2 (VEGFR-2) Y1175 [13]. Three types of VEGFR exist; VEGFR-2 plays a vital role in angiogenesis [14–16]. Phosphorylation of VEGFR-2 Y1175 is essential for promoting EC migration and angiogenesis. When VEGFR-2 Y1175 is normally phosphorylated, downstream signals including PLC/PKC/MAPK, PI3K, Akt, and Src are further activated to promote EC proliferation and migration [17–19]. However, few reports are available on the role of TULA-2/SYK/VEGFR-2 signal transduction pathways in angiogenesis, and no reports are available on the signal transduction pathways involved in aging-related angiogenesis disorders to our knowledge. Our study showed that the expression level of miR-25-3p decreased in aging mouse ECs through gene microarray in a previous study [7]. To reveal the specific roles of miR-25-3p in angiogenesis, we investigated the expression profiles of miR-25-3p and its target protein TULA-2 *in vitro*, establishing an acute lower limb ischemia model in aged mice. We found that miR-25-3p promotes angiogenesis in senescent ECs *via* TULA-2/SYK/VEGFR-2 signaling transduction *in vitro* and *in vivo*.

## RESULTS

### miR-25-3p expression in old endothelial cells (OECs) is downregulated

**Figure 1:**
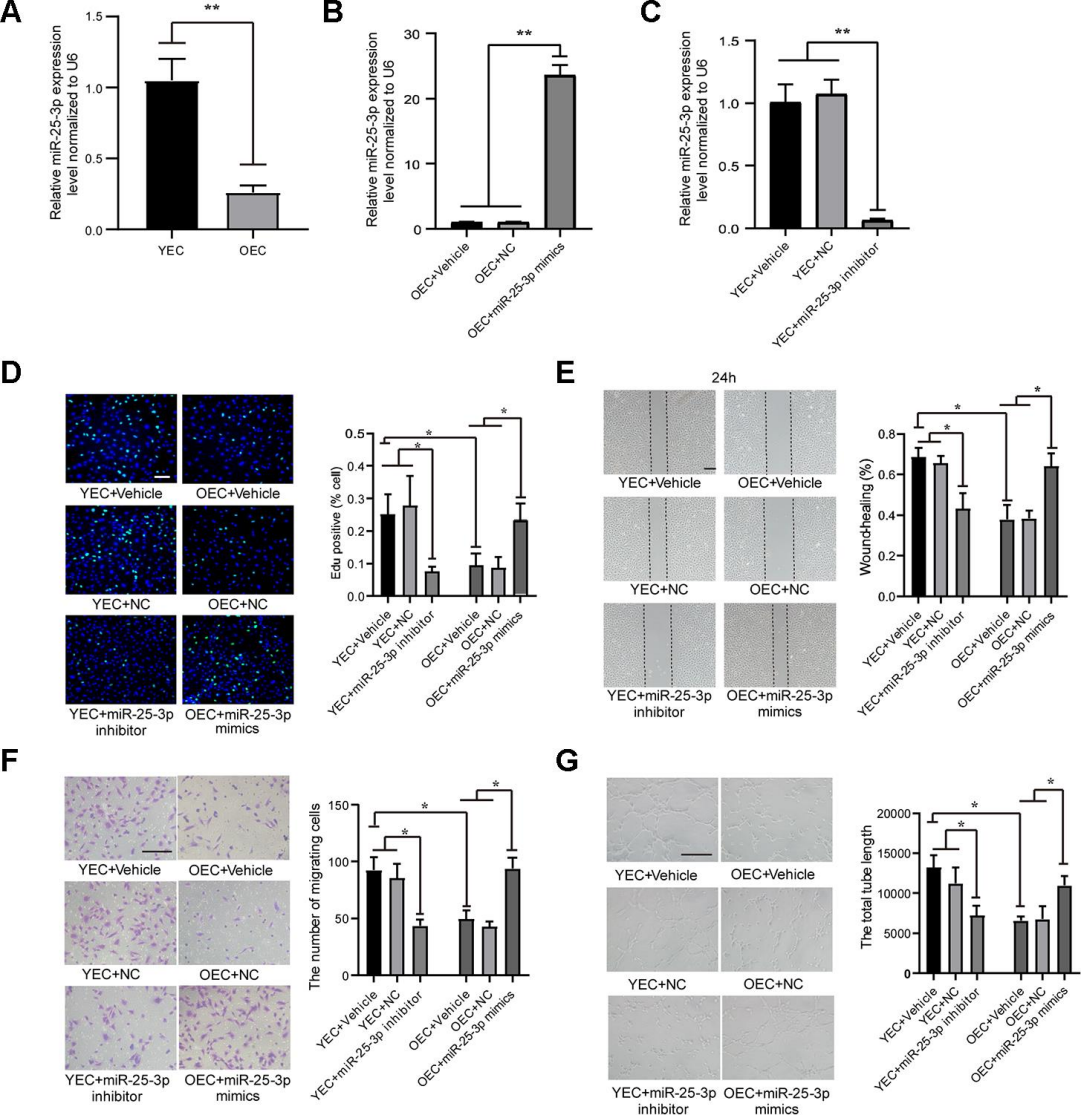
**MiR-25-3p expression in ECs: transfection with an miR-25-3p mimic or inhibitor regulates angiogenesis *in vitro*.** (**A**) RT-qPCR analysis showed that the expression of miR-25-3p was downregulated in old endothelial cells (OECs) versus young endothelial cells (YECs) (n=3). (**B**) RT-qPCR analysis showed that the expression of miR-25-3p was upregulated in OECs with miR-25-3p mimics(n=3). (**C**) RT-qPCR analysis showed that the expression of miR-25-3p was downregulated in YECs with miR-25-3p inhibitor(n=3). (**D**) An EdU assay showed that mouse EC proliferation was increased by the transfection of miR-25-3p mimics into OECs, whereas the miR-25-3p inhibitor showed the opposite effect in YECs (n=5; Scale bar, 100 μm). (**E**) A wound-healing assay showed that miR-25-3p mimics promoted the migration ability of OECs, whereas the miR-25-3p inhibitor showed the opposite effect in YECs (n=4; scale bar, 200 μm). (**F**) Transwell assays showed that miR-25-3p mimics promoted the migration ability of OECs, whereas the miR-25-3p inhibitor showed the opposite effect in YECs (n=5; scale bar, 100 μm). (**G**) Tube formation determined on Matrigel showed that miR-25-3p mimics increased the total tube length of OECs, whereas the miR-25-3p inhibitor showed the opposite effect in YECs (n=4; Scale bar, 100 μm) (*P < 0.05, **P < 0.01).

Our previous studies showed that the level of miR-25-3p decreased in OECs, as demonstrated by gene microarray. To further verify differences in miR-25-3p associated with aging, we extracted ECs from young (8 weeks) and old (12 months) C57bl/6 mice. Total RNA was extracted, and the real-time quantitative polymerase chain reaction (RT-qPCR) results showed that the expression of miR-25-3p in OECs was significantly downregulated compared with that in young endothelial cells (YECs) (Figure 1A).

### miR-25-3p promotes angiogenesis *in vitro*

To reveal the role of miR-25-3p in angiogenesis *in vitro*, we first proved that miR-25-3p can be efficiently overexpressed (Figure 1B) or inhibited (Figure 1C) by transfection with miR-25-3p mimics or miR-25a-3p inhibitors, respectively. Then, changes in angiogenesis were evaluated by EdU, wound-healing, transwell and tube formation assays. We inhibited miR-25-3p by transfecting 100 nM miR-25-3p inhibitors into YECs and found that YEC proliferation (Figure 1D), migration (Figure 1E, 1F) and tube formation (Figure 1G) were significantly lower than those in the control group. We overexpressed miR-25-3p by transfecting 50 nM miR-25-3p mimics into OECs and found that the proliferation (Figure 1D), migration (Figure 1E, 1F), and tube formation (Figure 1G) of OECs were significantly increased.

### TULA-2 protein expression is increased in OECs and reduces the phosphorylation level of SYK Y323 and VEGFR2 Y1175

**Figure 2:**
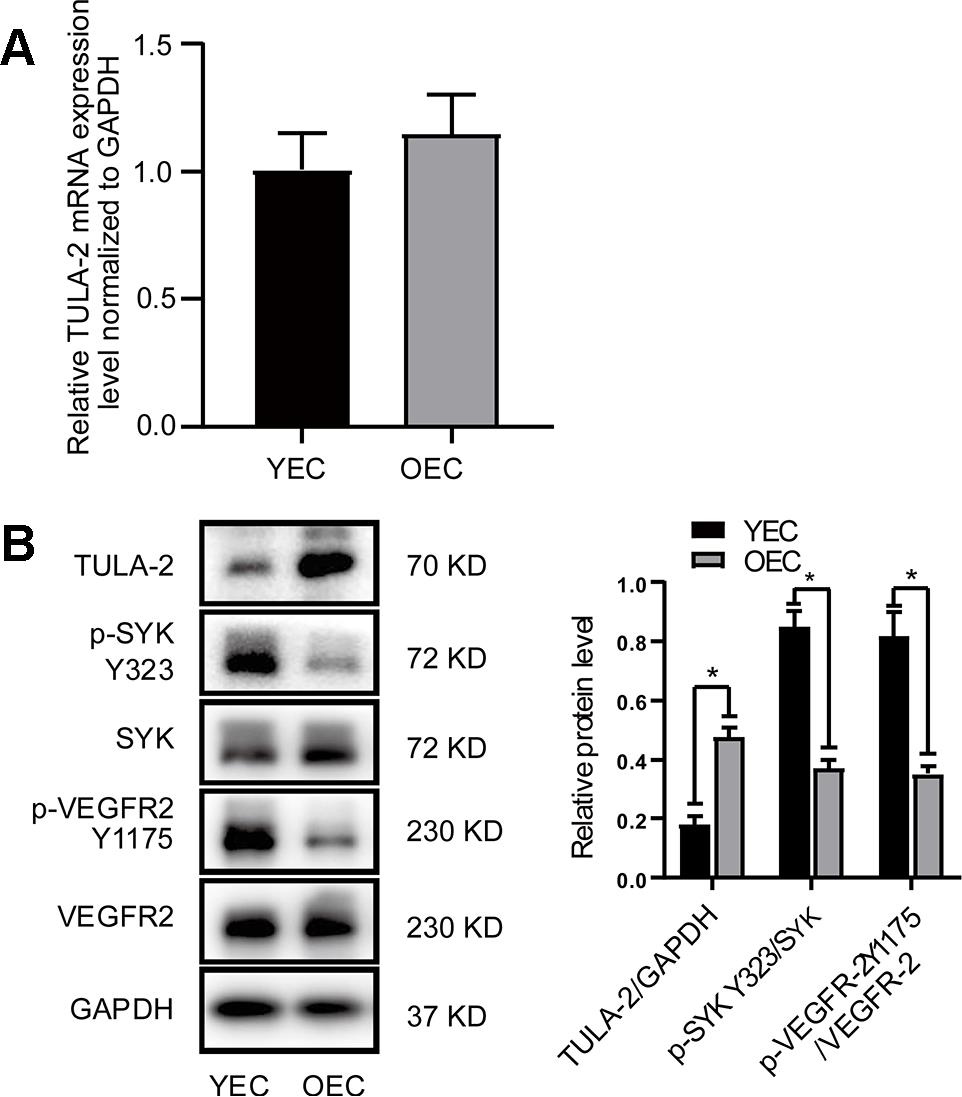
**Protein and mRNA expression of TULA-2, SYK Y323 and VEGFR-2 Y1175 in ECs.** ECs were incubated under normal culture conditions. (**A**) RT-qPCR showed no significant change in the mRNA levels of TULA-2 in OECs. (**B**) Western blot analysis revealed an increase in the protein levels of TULA-2 in OECs, and the phosphorylation levels of SYK Y323 and VEGFR-2 Y1175 were decreased in OECs (*P < 0.05, n = 3).

We evaluated the expression profiles of the TULA-2 gene and protein in YECs and OECs by RT-qPCR and Western blot, respectively. Our data showed no significant difference in TULA-2 mRNA expression between the two groups (Figure 2A). The expression of TULA-2 protein in OECs was higher than that in YECs (Figure 2B). TULA-2 can reduce the phosphorylation level of SYK Y323 by acting as a PTP [10, 11]. In addition, the decrease in SYK Y323 phosphorylation can reduce the phosphorylation of VEGFR-2 Y1175 and suppress angiogenesis [13]. Interestingly, our study showed that the downstream targets of TULA-2, p-SYK Y323 and p-VEGFR-2 Y1175 exhibited decreased protein expression in OECs compared to YECs (Figure 2B).

### MiR-25-3p targets TULA-2

**Figure 3:**
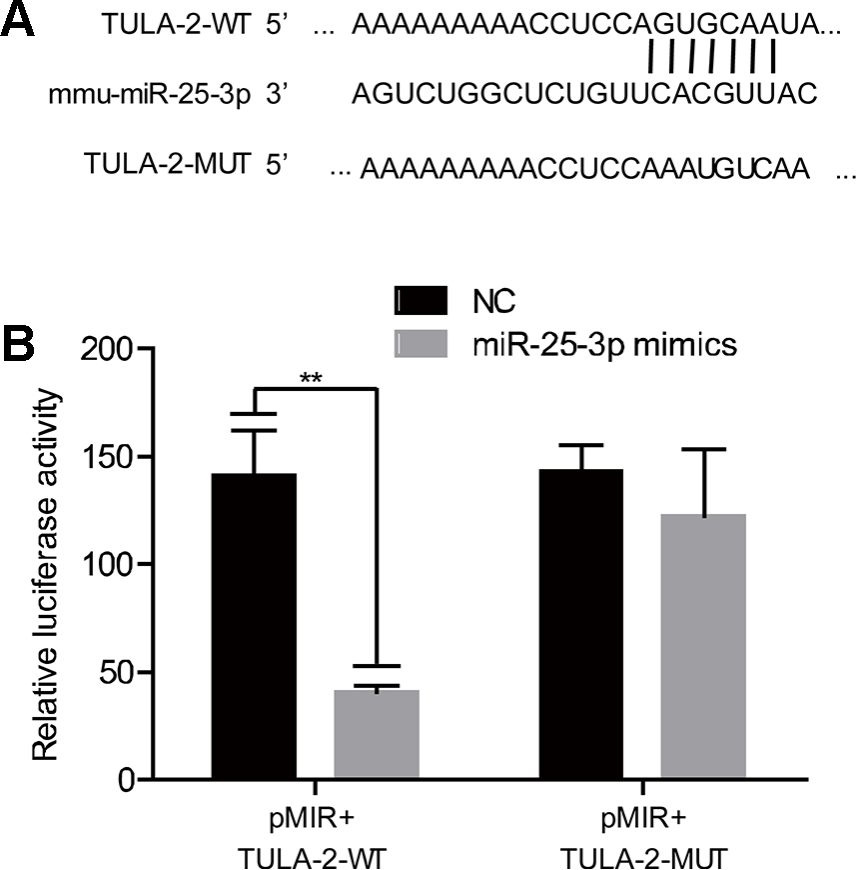
**TULA-2 is the downstream target of miR-25-3p.** (**A**) The predicted and conserved miR-25-3p target sites in the 3’UTR of TULA-2. (**B**) The luciferase activity of the WT 3’UTR but not the mutated 3’ UTR of TULA-2 was downregulated by miR-25-3p mimics in ECs (**P < 0.01 versus the negative control).

To investigate the details of the mechanism of miR-25-3p in regulating angiogenesis, we searched for miR-25-3p targets using miRNAMap and TargetScan databases and found target protein TULA-2. We found that the 3'UTR 240-246 sites of TULA-2 were complementary to miR-25-3p (Figure 3A). A Dual-luciferase assay showed that miR-25-3p significantly reduced the 3'UTR activity of TULA-2, which was manifested by weakened luciferase activity (Figure 3B). However, miR-25-3p did not decrease the 3'UTR activity of the mutant form of TULA-2, which lost its capability to target and bind to miR-25-3p. These results suggest that TULA-2 was a target of miR-25-3p. Targeted binding of miRNAs to RNA leads to translation blockade or degradation of RNA, which would affect the expression of TULA-2 mRNA. However, we found no significant change in TULA-2 mRNA expression profiles between YECs and OECs (Figure 2A), suggesting that regulation of miR-25-3p is achieved by translational inhibition rather than mRNA synthesis/degradation.

### MiR-25-3p positively regulates the phosphorylation of angiogenesis-related factors

As shown above, miR-25-3p can promote angiogenesis *in vitro*, and its expression level is inversely related to TULA-2 levels in ECs. Dual-luciferase assays showed that TULA-2 is a direct target of miR-25-3p.

**Figure 4:**
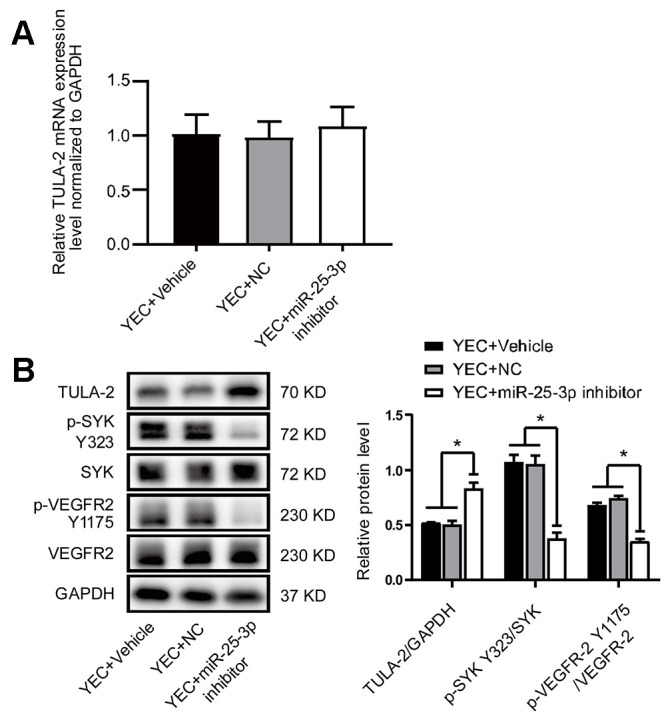
**The miR-25-3p inhibitor negatively regulates the angiogenic signaling pathway by reducing angiogenic growth factor expression.** (**A**) RT-qPCR showed no significant change in the TULA-2 mRNA level in YECs after transfection with the miR-25-3p inhibitor. (**B**) Western blot analysis showed that the miR-25-3p inhibitor upregulated the protein level of TULA-2 and downregulated the phosphorylation levels of SYK Y323 and VEGFR-2 Y1175 in YECs (n=3, data are expressed as the mean ± SEM, *P < 0.05 versus the negative control).

To further explore the function of miR-25-3p and elucidate the relationship between miR-25-3p and TULA-2, we first transfected an miR-25-3p inhibitor into YECs and evaluated the expression profiles of TULA-2, SYK, SYK Y323, VEGFR-2 and VEGFR-2 Y1175 by Western blot assays. We found no change in TULA-2 mRNA in YECs (Figure 4A). However, after miR-25-3p was downregulated, TULA-2 protein expression increased, SYK Y323 and VEGFR-2 Y1175 phosphorylation decreased, and the total protein levels of SYK and VEGFR-2 did not change significantly (Figure 4B), indicating that the phosphorylation of angiogenetic growth factors can be reduced by downregulating miR-25-3p in YECs.

**Figure 5:**
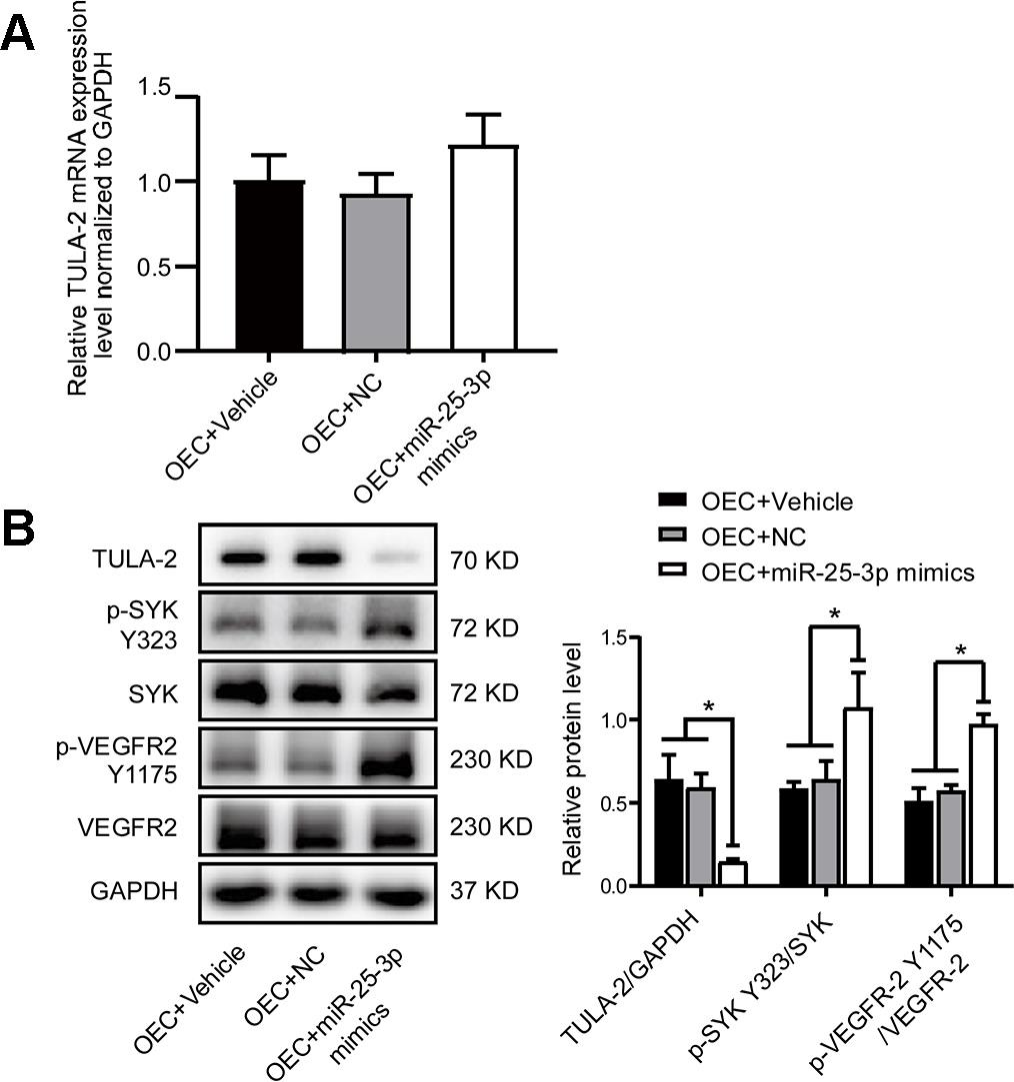
**MiR-25-3p mimics positively regulate the angiogenic signaling pathway by increasing angiogenic growth factor expression.** (**A**) RT-qPCR showed no significant change in the TULA-2 mRNA level in OECs after transfection with miR-25-3p mimics. (**B**) Western blot analysis showed that miR-25-3p mimics downregulated the protein level of TULA-2 and upregulated the phosphorylation levels of SYK Y323 and VEGFR-2 Y1175 in OECs (n=3, data are expressed as the mean ± SEM, *P < 0.05 versus the negative control).

We transfected miR-25-3p mimics into OECs and found no significant difference in TULA-2 mRNA (Figure 5A) but instead noted increased phosphorylation levels of SYK Y323 and VEGFR-2 Y1175 (Figure 5B), indicating that miR-25-3p upregulation in OECs can increase the phosphorylation of angiogenetic growth factors.

These results revealed that miR-25-3p can modulate the phosphorylation of SYK Y323 and VEGFR-2 Y1175 *via* TULA-2, thus regulating changes in endothelial-mediated angiogenesis.

### TULA-2 knockdown promotes angiogenesis

**Figure 6:**
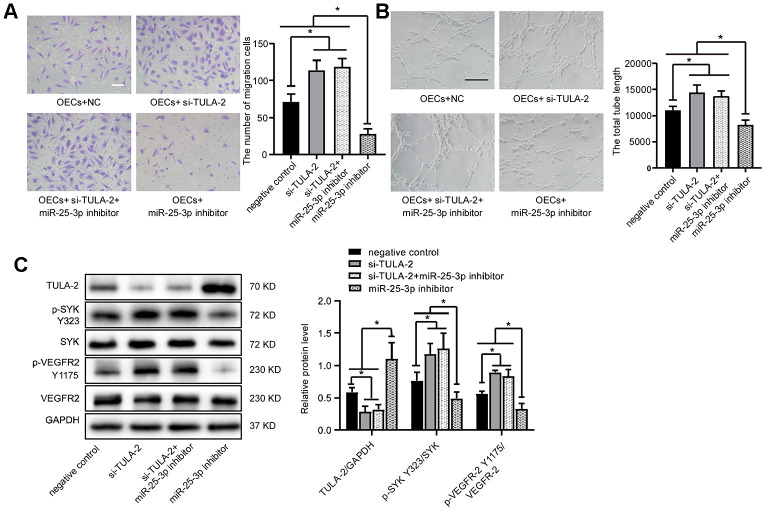
**TULA-2 knockdown positively regulates angiogenesis *in vitro* and increases p-SYK Y323 and p-VEGFR-2 Y1175 expression at the protein level.** (**A**) Transwell assays showed that TULA-2 siRNA promoted the migration ability of OECs, and co-transfection of OECs with both si-TULA-2 and miR-25-3p inhibitors did not suppress the effect of TULA-2 upregulation on EC migration relative to the effects of siRNA-NC (n=5; Scale bar, 100 μm). (**B**) Tube formation determined on Matrigel showed that TULA-2 siRNA increased the total tube length, and co-transfection of OECs with both si-TULA-2 and miR-25-3p inhibitors did not suppress the effect of TULA-2 upregulation on EC tube formation relative to the effects of siRNA-NC (n=4; scale bar, 100 μm). (**C**) Western blot analysis of relative TULA-2, SYK Y323 and VEGFR-2 Y1175 expression in OECs transfected with the negative control, si-TULA-2, si-TULA-2+ miR-25-3p inhibitor and miR-25-3p inhibitor (data are expressed as the mean ± SEM, *P < 0.05, **P < 0.01 versus the negative control).

To study whether miR-25-3p can promote angiogenesis through TULA-2, siRNA was used to knock down TULA-2 in OECs. The results showed that compared with the control group, the migration ability and tube formation ability were significantly improved as evidenced by transwell and tube formation assays, respectively, but co-transfection with si-TULA-2 and miR-25-3p inhibitors did not suppress the effect of si-TULA-2 (Figure 6A, 6B). The effect of TULA-2 knockdown on angiogenesis was similar to that of miR-25-3p overexpression, and the phosphorylation levels of SYK Y323 and VEGFR-2 Y1175 were increased, but co-transfection with si-TULA-2 and miR-25-3p inhibitors did not suppress this effect (Figure 6C), suggesting that miR-25-3p directly modulated the expression profile of TULA-2, affected the phosphorylation levels of SYK Y323 and VEGFR-2 Y1175, and regulated angiogenesis.

### The direct binding of TULA-2 and SYK may inhibit the phosphorylation of angiogenesis-related factors

**Figure 7:**
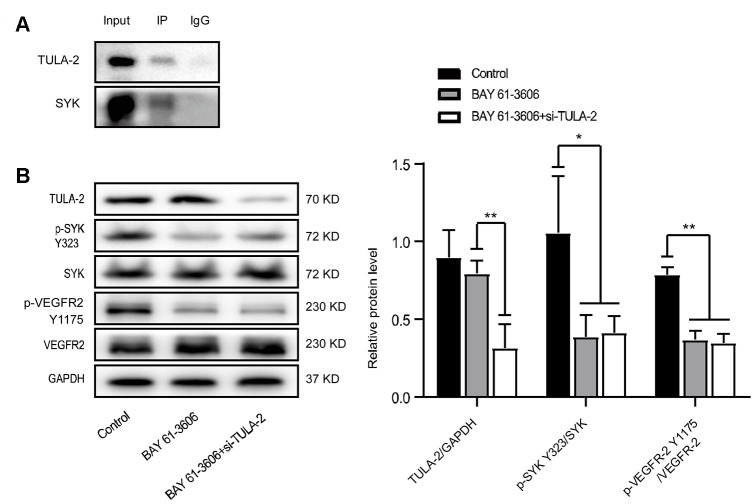
**The interaction of TULA-2 and SYK inhibits the phosphorylation of SYK Y323 and VEGFR-2 Y1175.** (**A**) Western blot analysis of the Co-IP experiment showed that SYK immunoprecipitated with anti-TULA-2, SYK and TULA-2, which was visualized by Western blot. (**B**) Western blot analysis of relative TULA-2, SYK Y323 and VEGFR-2 Y1175 expression in YECs transfected with the control, BAY 61-3606 (SYK inhibitor), and BAY 61-3606 + si-TULA-2. The phosphorylation levels of SYK Y323 and VEGFR-2 Y1175 were decreased in YECs after transfection with BAY 61-3606 or BAY 61-3606 + si-TULA-2 (data are expressed as the mean ± SEM, *P < 0.05 versus the negative control).

We found a direct combination of TULA-2 and SYK in ECs through co-immunoprecipitation (Co-IP) (Figure 7A). We incubated ECs with an SYK phosphorylation inhibitor (BAY 61-3606). The phosphorylation levels of SYK Y323 and VEGFR-2 Y1175 were significantly decreased, while the expression of TULA-2 did not change. Next, we incubated ECs with BAY 61-3606 and si-TULA-2. The protein expression profile of TULA-2 decreased significantly, but the phosphorylation levels of SYK Y323 and VEGFR-2 Y1175 were not significantly changed compared with those in ECs incubated with BAY 61-3606 (Figure 7B). These results suggested that si-TULA-2 and SYK phosphorylation inhibitors have opposite effects, and that TULA-2 binding with SYK may inhibit the phosphorylation of SYK Y323 in ECs, thus affecting the phosphorylation of VEGFR-2 Y1175.

### Upregulation of miR-25-3p promotes the recovery of acute hindlimb ischemia in aging mice

**Figure 8:**
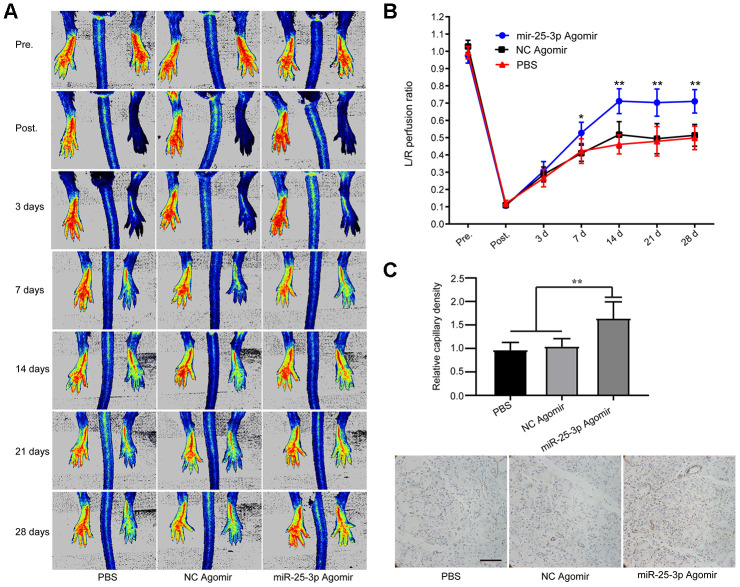
**MiR-25-3p can promote ischemia-initiated blood flow recovery and angiogenesis in aged mice.** (**A**) Representative images of laser Doppler blood flow before, immediately after, and 3, 7, 14, 21 and 28 days after femoral artery resection. (**B**) Blood flow in the ischemic hind limb was measured. The results are expressed as a ratio of the perfusion in the left limb (ischemic) to that in the right limb (control, nonischemic). The recovery of lower limb blood flow in the miR-25-3p agomir group (n=5) was better than that in the NC agomir group (n=5) and the PBS group (n=5) on the 7^th^, 14^th^ and 28^th^ days. (**C**) Representative images of anti-CD31 immunohistochemical sections of the gastrocnemius muscle of the left lower extremity. The expression level of CD31 in the miR-25-3p agomir group was higher than those in the NC agomir and PBS groups. For each animal, 5-6 randomly selected fields from 3-5 sections were counted (*P < 0.05, **P<0.01).

To further verify the role of miR-25-3p *in vivo*, we established an acute hindlimb ischemia model in aged mice (C57blc/6, 12 months old) and randomly divided the mice into three groups (see experimental materials and methods). MiR-25-3p agomir, PBS or NC agomir were injected into the tail vein on the first day before surgery and 1 day and 3 days after surgery. We found that blood perfusion in the ischemic lower limbs in the miR-25-3p agomir group was significantly better than that in the NC agomir group and the PBS group on days 3, 7, 14, 21, and 28 (Figure 8A, 8B), indicating that upregulation of miR-25-3p can promote the recovery of lower limb ischemia in aged mice.

To confirm the pathological changes in the ischemic tissues, we performed immunohistochemical staining. Based on the key role of arteriogenesis in the recovery of blood perfusion, we determined the degree of arteriogenesis by measuring vascular density. Sections of ischemic gastrocnemius muscle tissue from mice were prepared 14 days after femoral artery ligation, and immunohistochemical staining was performed with an anti-CD31 antibody. The results showed that the expression of CD31 in the miR-25-3p agomir group was significantly higher than that in the NC agomir group and the PBS group, and no significant difference was found between the NC agomir group and the PBS group (Figure 8C). The results showed that upregulation of miR-25-3p levels promoted capillary regeneration in ischemic tissues of lower limbs in aged mice.

**Figure 9:**
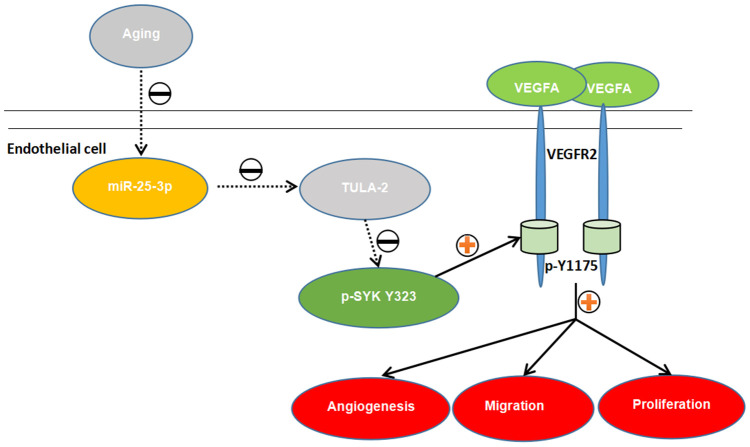
**The proposed novel mechanism by which miR-25-3p affects angiogenesis in aged mice *via* the TULA-2/SYK/VEGFR-2 pathway.** Aged ECs utilize miR-25-3p to target TULA-2 as a downstream effector to transduce signals, further downregulating and suppressing the phosphorylation of SYK Y323 and VEGFR-2 Y1175 and thus suppressing the angiogenesis pathway and inhibiting the proliferation, migration and tube formation abilities of aged ECs.

The above experiments demonstrated that the miR-25-3p/TULA-2/SYK/ VEGFR-2 signal transduction pathway exists in ECs. The binding of TULA-2 with SYK may inhibit the phosphorylation of SYK Y323 in ECs, and this process affects the phosphorylation of VEGFR-2 Y1175, thus regulating angiogenesis (Figure 9).

## DISCUSSION

In our study, we found that miR-25-3p was significantly downregulated in OECs. Further experiments showed that miR-25-3p can participate in EC proliferation, migration and tube formation by targeting TULA-2 and its downstream SYK/VEGFR-2 signaling pathway, thereby affecting EC-mediated vascular regeneration.

Recent studies have revealed that miRNAs are widely involved in the regulation of intracellular signaling pathways, including the differential expression of angiogenesis-related proteins during aging [6]. As aging occurs, angiogenic potential declines, which is related to endothelial dysfunction, and VEGFR-2 plays an essential role in angiogenesis. Qun [20] found no difference in the expression of VEGFR-2 between old and young mouse ECs but that the phosphorylation level of VEGFR-2 in young mice was significantly higher than that in old mice after ischemia; thus, the activity of the signal transduction pathway of vascular ECs declined with age. In addition, miRNA can inhibit the expression of negatively regulated target genes *via* degradation or translation. Increasing evidence indicates that miRNAs in ECs play a pivotal role in the molecular mechanisms of angiogenesis in aging animals [7, 21, 22]. Relevant studies have found that the expression profiles of miR-142-3p, miR-146, miR-29 and miR-223 were significantly increased in the aortas of aging mice [21], and that miR-21, miR-217, miR-216, miR-31b, miR-181b and miR-34a play pivotal roles in EC age regulation. [23]. Our previous gene microarray screening showed that the expression profile of miR-25-3p was decreased in OECs, and RT-qPCR further confirmed the downregulated miR-25-3p expression in OECs.

To date, research on miR-25-3p has mostly focused on cancer. Increased miR-25-3p expression can be used as a diagnostic marker for invasive osteosarcoma [24] and can promote the migration and invasion of many tumors, such as liposarcoma [25] and cholangiocarcinoma [26]. In rectal cancer, miR-25-3p can promote the formation of a pre-metastatic niche by inducing angiogenesis [27]. MiR-25-3p in human cancer cells promotes angiogenesis by enhancing EC proliferation and migration, thereby promoting tumor migration and invasion [27]. In our study, we found that upregulation of miR-25-3p in OECs can promote the migration, proliferation and tube formation of OECs, while downregulation of miR-25-3p in YECs can inhibit the migration, proliferation and tube formation of YECs. This is the first demonstration that miR-25-3p is related to cellular aging and can promote angiogenesis.

We predicted the target of miR-25-3p using the TargetScan and miRNA Map databases and found that the TULA-2 protein may be a potential target. TULA-2 can negatively regulate the phosphorylation level of SYK Y323 by acting as a histidine PTP [10, 11], while downregulation of the phosphorylation level of SYK Y323 can inhibit the phosphorylation of VEGFR-2 Y1175 [13]. Interestingly, in our study, we found that the TULA-2 expression profile was upregulated, while the phosphorylation levels of SYK Y323 and VEGFR-2 Y1175 were downregulated in OECs compared with YECs. Thus, miR-25-3p can play a key role in regulating angiogenesis *via* the TULA-2/SYK/VEGFR-2 signal transduction pathway.

We confirmed that the TULA-2 gene is a direct target of miR-25-3p by luciferase assay. To investigate the relationship between miR-25-3p and TULA-2, miR-25-3p mimics were overexpressed in OECs. We found that the expression profile of TULA-2 was downregulated, while the phosphorylation levels of SYK Y323 and VEGFR-2 Y1175 were upregulated. In YECs, the expression level of TULA-2 was upregulated, and the phosphorylation levels of SYK Y323 and VEGFR-2 Y1175 were downregulated after transfection with miR-25-3p inhibitors, suggesting that miR-25-3p may affect the phosphorylation of SYK Y323 and VEGFR-2 Y1175 sites by regulating TULA-2 levels, thereby positively regulating angiogenesis.

To verify whether miR-25-3p promotes angiogenesis by targeting TULA-2, we used siRNA to knock down the expression profile of TULA-2 in ECs and found that EC migration and tube formation were enhanced. Western blot analysis suggested that the phosphorylation levels of SYK Y323 and VEGFR-2 Y1175 were increased, which was associated with overexpression of miR-25-3p. This result showed that defects in angiogenesis are related to the elevated levels of TULA-2, and that knocking down the expression of TULA-2 can promote angiogenesis.

To further explore the relationship between TULA-2 and its downstream proteins, we used Co-IP and found a direct association between TULA-2 and SYK in ECs. We used an SYK phosphorylation inhibitor (BAY 61-3606) to inhibit the phosphorylation of TULA-2 and found that the phosphorylation level of VEGFR-2 Y1175 was decreased. The results indicated that the presence of TULA inhibited the phosphorylation of SYK Y323 by directly binding to SYK and affected the phosphorylation of VEGFR-2 Y1175. Previous research confirmed this finding [10, 11, 13]. All the above studies proved the existence of the TULA-2/SYK/VEGFR-2 signaling pathway in ECs during aging, which is regulated by miR-25-3p and plays a crucial role in angiogenesis (Figure 9).

Experiments *in vitro* have shown that miR-25-3p can downregulate the expression profile of TULA-2 and promote the angiogenesis of OECs. Recovery of blood perfusion after ischemia mainly depends on capillary regeneration, which is closely related to the proliferation and migration of vascular ECs [28]. To verify the function of miR-25-3p in promoting angiogenesis *in vivo*, we established an acute lower limb ischemia model in aged C57bcl/6 mice by ligating and severing the femoral artery. We found that after tail vein injection of miR-25-3p agomir, the recovery of blood perfusion in ischemic limbs and the density of new capillaries in the gastrocnemius muscle were better than those in the control group, suggesting that upregulation of miR-25-3p level *in vivo* can promote capillary regeneration and blood flow recovery in the lower extremities of aged mice with acute ischemia.

The VEGF-VEGFR2 signaling pathway is well known as a key angiogenesis signaling pathway [29]. Many studies [30–32] have shown that the VEGF-VEGFR2 signaling pathway is at the core of angiogenesis, which mainly depends on the combination of VEGF with VEGFR2 to subsequently affect the phosphorylation of VEGFR2, including the VEGFA/VEGFR2/FAK, mTOR/HIF1-α/VEGF, FOXF1/VEGFA pathways. To our knowledge, this study is the first to demonstrate the presence of an miR-25-3p-mediated TULA-2/SYK/VEGFR2 signaling pathway in OECs and showed that the phosphorylation of VEGFR2 may also be affected by another different pathway in which miR-25-3p plays an important role. These findings further complement the VEGF-mediated angiogenesis pathway and provide new approaches for future angiogenesis treatment in the aging population.

However, some limitations exist in this study. The method by which age affects miR-25-3p levels, whether miR-25-3p affects angiogenesis through other targets, and the intrinsic mechanism by which TULA-2 affects SYK levels and the phosphorylation of VEGFR-2 require further extensive research.

In summary, we elucidated the pivotal role of miR-25-3p in regulating angiogenesis *via* TULA-2 *in vitro* and *in vivo*. This study suggested that increasing miR-25-3p levels can promote the regeneration of aging blood vessels *via* the TULA-2/SYK/VEGFR-2 signaling pathway. Although many theoretical and technical issues remain to be resolved, miR-25-3p and TULA-2 may be promising therapeutic targets for cardiovascular diseases.

## MATERIALS AND METHODS

### Cell culture and transfection

Arterial ECs were obtained from the thoracic aorta of C57bl/6 male mice and cultured using endothelial growth medium-2 (EGM-2, LONZA, USA) plus 10% fetal bovine serum (FBS, Thermo Fisher Scientific, USA) and antibiotics (penicillin 100 IU/ml and streptomycin 100 μg/ml, Thermo Fisher Scientific) at 37°C in a humidified incubator (Thermo Fisher Scientific) with 5% CO_2_. Each cluster of cells was collected from three mice in the same feeding batch for each experiment, and cells at passages 3–5 were used in this study. All murine studies conformed to the Declaration of Helsinki and received approval from the Clinical Research and Experimental Animal Ethics Committee of the First Affiliated Hospital Sun Yat-sen University.

Transfection with miR-25-3p mimics, an miR-25-3p inhibitor and si-TULA-2 was conducted using Lipofectamine 3000 (Thermo Fisher Scientific), in a 5% CO_2_ and 37°C cell incubator, using serum-free medium for 6 hours, then changing to 10% serum medium. The transfection concentrations of miR-25-3p mimics, the miR-25-3p inhibitor and si-TULA-2 were 50 nM, 100 nM and 50 nM, respectively, according to the specific instructions (RiboBio Guangzhou, China). miR-25-3p mimic 5'-CAUUGCACUGUCUCGGUCUGA-3', miR-25-3p inhibitor 5'-CAUUGCACUUGUCUCGGUCUGA-3', si-TULA-2 5'-CCCAGAACAUUGACGUCAATT-3', and si-NC 5'-UUCUCCGAACGUGUCACGUTT-3' were synthesized by RiboBio. Then, follow-up experiments were carried out according to the experimental design. Generally, RNA can be extracted after 24 hours, and the total protein of cells can be extracted after 48-72 hours.

### Quantitative real-time polymerase chain reaction

Each group of ECs was collected, and total RNA was extracted using Trizol reagent (Invitrogen, USA). A single RNA sample was reverse transcribed into cDNA using the transcript first strand cDNA synthesis kit (Takara, Dalian, China). The relative mRNA transcription levels of the target gene were determined by RT-qPCR using specific primers on the FastStart Universal SYBR Green Master (Takara) and Roche LightCycler 480 Real-Time PCR systems. The sequences of the primers were as follows: forward 5’- TCTCCCGACATTCACCCTG-3’ and reverse 5’- TTCAACACACATCACTCGTTCTCT-3’ for TULA-2, forward 5’- ACACTCCAGCTGGGCATTGCACTTGTCTCG-3’ and reverse 5’- ACACTCCAGCTGGGCATTGCACTTGTCTCG -3’ for miR-25-3p, forward 5’-AAGTATTTC GATTTCTTGGC-3’ and reverse 5’-AATATGGAACGCTTCACG-3’ for U6, and forward 5’- AGGTCGGTGTGAACGGATTTG -3’ and reverse 5’ - TGTAGACCATGTAGTTGAGGTCA -3’ for GAPDH. U6 and GAPDH served as reference genes for the detection of miR-25-3p and TULA-2, respectively. PCR was repeated three times, and the results were calculated using the 2^-ΔΔCt^ method.

### Western blot

Western blot was used to detect the relative expression of TULA-2, SYK, p-SYK Y323, VEGFR-2 and p-VEGFR-2 Y1175. The ECs were incubation normal culture conditions for 48-72 hours and then ice-lysed in RIPA buffer (Thermo Fisher Scientific, Inc.), and the Pierce BCA Protein Assay Kit (Thermo Scientific, USA) was used to measure the protein concentration. Equal amounts (20 μg) of protein were separated by 10% SDS-PAGE and transferred to a polyvinylidene fluoride (PVDF) membrane (Millipore, Germany). After blocking with TBST supplemented with 5% bovine serum albumin (BSA) (BioFroxx Inc, Germany), the PVDF membrane was incubated at 4°C overnight with primary antibodies against mouse TULA-2 (1:500; cat. no. sc-514612; Santa Cruz, CA, USA), SYK (1:1000; cat. no. 13198; Cell Signaling Technologies, Beverly, USA), p-SYK Y323 (1:1000; cat. no. 2715; Cell Signaling Technologies), VEGFR-2 (1:1000; cat. no. 9698; Cell Signaling Technologies), p-VEGFR-2 Y1175 (1:1000; cat. no. 2478; Cell Signaling Technologies) and GAPDH (1:1000; cat. no. 5174; Cell Signaling Technologies). The corresponding secondary antibodies of anti-mouse horseradish peroxidase (HRP)-conjugated secondary antibody (1:6000; cat. no. 58802) and anti-rabbit HRP-conjugated secondary antibody (1:6000; cat. no. 93702). were purchased from Cell Signaling Technologies. Detection was performed using Novex ECL (Invitrogen), and signal quantification was performed using ImageJ software (version 1.8.0).

### Co-immunoprecipitation (co-IP)

The extraction method for total protein refers to the above Western blot experiments. An equal amount of protein (200-300 μg) was used for a lysate preclearing step (1 h at 4°C) using a 30 μl Protein G Plus/Protein A Agarose Suspension (Millipore, IP05). For immunoprecipitation (IP), precleared lysates were incubated with 3 μl of mouse anti-TULA-2 (cat. no. 514612; Santa Cruz), then another incubation with 30 μl of Protein G Plus/Protein A Agarose Suspension for 3 hours (at 4°C). For the control, the same amount of precleared lysates was incubated with mouse nonspecific IgG (cat. no. 2729; Cell Signaling Technologies) covalently coupled with Protein G Plus/Protein A Agarose Suspension. After supernatants were removed, beads were washed with IP lysis buffer, and samples were analyzed *via* Western blotting.

### 3’-UTR luciferase assays

A mutant version of TULA-2 within the miR-25-3p binding sequence was obtained using the QuikChange II Site-Directed Mutagenesis Kit (Stratagene, USA). The wt and mut pmiR-RB-REPORT dual-luciferase vectors of TULA-2 (wt-TULA-2 and mut-TULA-2, respectively) were designed by RiboBio Biotech Co., Ltd. An NC mimic or miR-25-3p mimics was co-transfected with wt-TULA-2 or mut-TULA-2 into 293T cells in 24-well plates using Lipofectamine 3000 (Thermo Fisher Scientific). After 24 hours of transfection, a Dual-Luciferase Reporter Assay System Kit (Promega Biotech, USA) was used to detect luciferase efficiency. All the experiments were repeated three times.

### *In vitro* wound-healing assay

ECs were plated in 6-well plates at 90% confluence after transfection with miRNA or siRNA. Using a sterile 1-ml pipette tip to create a wound scratch relative to the reference point. After washing the cells with the appropriate media, the first phase-contrast image was acquired by a light microscope (Olympus Co., Ltd.) based on the reference point. Subsequently, the cells were incubated with 1% FBS EGM-2 medium for 24 hours, and a second image at the same region was acquired. The area of the wounded region without cells was measured using ImageJ software (version 1.8.0).

### Transwell assay

Transwells with an 8-μm-pore membrane were inserted into well plates (Corning Life Sciences, USA). Next, 10% FBS EGM-2 was first added under the chamber, and then the treated ECs were cultured in the upper chamber with serum-free EGM-2 for 24 hours. Then, the chambers were fixed in 4% paraformaldehyde for 20 minutes and then stained with crystal violet for 15 min. After the upper surface of the inserted transwell membrane was wiped clean, the migrated cells were counted in five random regions using a light microscope (Olympus Co., Ltd., Japan).

### Tube formation

The treated cells were seeded in 96-well plates precoated with 100 uL of Matrigel® Growth Factor Reduced (GFR) Basement Membrane (Corning Life Sciences) under normal culture conditions. After 8 hours of incubation, photographs were taken with a light microscope (Olympus Co., Ltd.). The lengths of formed tubes were measured using ImageJ software (Version 1.8.0).

### Cell proliferation evaluation with EdU assay

The treated ECs were seeded in 96-well plates and incubated under normal culture conditions. When the fusion degree between cells reached 60-70%, ECs were labeled with a Cell-Light EdU Apollo488 Kit according to the specific protocol (Guangzhou RiboBio Co., Ltd.). Then, the proportion of EdU-positive cells was detected to assess the proliferation ability by fluorescence microscopy (BX51W; Olympus Co., Ltd.).

### Animal model

The C57bl/6 mice (male, 12 months) used in the experiment were obtained from Charles River (China). The mice were randomly divided into three groups: the agomir miR-25-3p group, the agomir NC group and the phosphate-buffered saline (PBS) group. Surgery was carried out under continuous inhalation of isoflurane anesthesia, and a 1-1.5-cm-long incision was made in the direction of the femoral artery in the groin area. The femoral artery was visualized, the subcutaneous tissue and femoral artery sheath were dissociated, and the femoral nerve and femoral artery were successively separated. Double ligation was performed at the distal end of the branch of the profunda femoris. After ligation, the femoral artery between the two ligation points was cut off, and then the skin was closed by suture. Tail vein injections of agomir miR-25-3p (80 mg/kg/day, 0.2 ml), agomir NC (80 mg/kg/day, 0.2 ml) and PBS (0.2 ml/day) were given 1 day before surgery and 1 day and 3 days after surgery. The study was conducted in accordance with the Guide to Animal Experiments of our institution.

### Blood flow measurements

Blood flow measurements were conducted preoperatively and 0, 3, 7, 14, 21, and 28 days after surgery by PeriCam PSI (Perimed, Germany) in a thermostatic and soundproof environment (room temperature 24, no glare, no noise). Using PIMSoft (Perimed, Sweden) software to analyze the image data. The ratio of ischemic limb blood flow to normal limb blood flow was used as an indicator of ischemic limb blood flow recovery.

### Capillary density measurement

Fourteen days after lower limb ischemia induction, the gastrocnemius muscle was excised and fixed with 4% paraformaldehyde for 24 h. After embedding in paraffin, the tissues were cut to 4-μm thickness for subsequent experiments. Histological sections were incubated with an anti-CD31 monoclonal antibody (1:300; cat. no. ab28364; Abcam, USA) as the first antibody, and anti-rabbit IgG (1:200; cat. no. 2357; Santa Cruz) was used as the secondary antibody. The sections were observed under a microscope and five fields were randomly selected for each section. CD31 positive regions were used as an indicator of capillary density.

### Statistics

All data are presented as the mean ± standard error of mean (SEM). Two treatment groups were compared using independent sample t-tests, three or more groups were compared using one-way analysis of variance (ANOVA), and multivariate comparisons were used to correct for minimal post hoc analysis (GraphPad Prism 8.0.1, USA). A *p*-value <0.05 was considered statistically significant.
